# Pak2 is essential for the function of Foxp3+ regulatory T cells through maintaining a suppressive Treg phenotype

**DOI:** 10.1038/s41598-017-17078-7

**Published:** 2017-12-06

**Authors:** Kyle L. O’Hagan, Stephen D. Miller, Hyewon Phee

**Affiliations:** 1 Department of Microbiology-Immunology, Feinberg School of Medicine, Northwestern University, Chicago, IL 60611 USA; 2 Department of Inflammation and Oncology, Amgen Inc., South San Francisco, CA 94080 USA

## Abstract

Foxp3, a key transcription factor that drives lineage differentiation of regulatory T cells (Tregs), was thought to imprint a unique and irreversible genetic signature within Tregs. Recent evidence, however, suggests that loss or attenuation of Foxp3 expression can cause Tregs to de-differentiate into effector T cells capable of producing proinflammatory cytokines. Herein, we report that the signaling kinase, p21-activated kinase 2 (Pak2), is essential for maintaining Treg stability and suppressive function. Loss of Pak2, specifically in Tregs, resulted in reduced expression of multiple Treg functional molecules, including Foxp3, CD25, Nrp-1 and CTLA-4, coupled with a loss of Treg suppressive function *in vitro* and *in vivo*. Interestingly, Pak2-deficient Tregs gained expression of Th2-associated cytokines and the transcription factor, Gata3, becoming Th2-like cells, explaining their inability to regulate immune responses. Collectively, these findings suggest Pak2 as an important signaling molecule for guarding against aberrant immune responses through regulating the stability of Foxp3^+^ Tregs and maintaining a suppressive Treg phenotype.

## Introduction

Defined by their expression of the X-linked transcription factor, Foxp3, naturally occurring regulatory T cells (Tregs) have been widely studied for their ability to induce self-tolerance and maintain peripheral immune homeostasis under multiple inflammatory settings^1–3^. Studies support that expression of Foxp3 is critical for the thymic development and suppressive capacity of Tregs, deeming Foxp3 as the master regulator of Treg biology^4–8^. Traditionally, it was thought that Foxp3 expression fully committed developing thymocytes to a terminally differentiated Treg lineage, with little evidence to support Treg plasticity and conversion to other T cell lineages^3^. However, more recent studies suggest that Tregs comprise a heterogeneous population of cells capable of expressing T helper (Th) cell lineage markers^3,9,10^. In fact, it has been proposed that the ability of Tregs to acquire expression of Th cell homing receptors and lineage-specific transcription factors, including T-bet, RORγt and Gata3, allows for more localized and fine-tuned suppressive control of respective effector responses^3^. In support of this, T-bet-deficient Tregs were inefficient in their proliferative response towards Th1-associated mycobacterium tuberculosis infection and trafficking to the inflamed lung^11^. A Treg-specific loss of *Gata3* resulted in the onset of spontaneous autoimmunity with an impaired ability to control the development of colitis^12,13^. Similarly, a Treg-specific loss of the Th2 differentiation factor, IRF-4, resulted in widespread multi-organ T and B cell autoimmunity^14^, suggesting that expression of Th2-associated genes may be important for Tregs to efficiently control local Th2 responses.

Given that Tregs are capable of expressing transcription factors that typically define Th subsets, it is surprising that Tregs do not de-differentiate and convert into cells of other T cell lineages. To this end, Foxp3 is thought to play a key role in maintaining this Treg identity and is responsible for regulating the complex interplay with Th cell transcription factors^10,15^. Indeed, several studies suggest that, upon losing or attenuating Foxp3 expression, Tregs lose their suppressive capacity and are capable of adopting a more proinflammatory phenotype that may exacerbate disease^3,15^. Using an elegant Foxp3 lineage tracing system, a small proportion of Tregs were shown to lose Foxp3 expression, even under homeostatic conditions^16^. This population, termed ‘ex-Tregs’, significantly increased in proportion in diabetic-prone NOD mice and exhibited an activated effector memory phenotype with the ability to produce IFN-γ and IL-17^16^. In line with this, deletion of Foxp3 in mature Tregs resulted in a loss of Treg suppressive function, *in vivo*, coupled with acquisition of Th1 and Th2 cytokine producing capabilities^17^. Interestingly, when Foxp3 expression was markedly reduced but not completely lost in the case of *FILIG* mice, a Th2-biased disease similar to that observed in Scurfy mice was observed^18^. This was a result of a loss of the characteristic Treg genetic signature as well as the conversion of Foxp3+ Tregs to IL4-producing Th2 cells^18^, collectively suggesting that Foxp3 expression levels directly control the balance of whether cells remain committed to the Treg lineage or become unstable and convert to alternative effector T cell lineages.

While the plasticity and stability of ‘committed’ Foxp3^+^ Tregs still remains controversial, the potential implications of this have become highly relevant with the interest and use of Treg-based therapies for autoimmune and other biological diseases^19^. Given that the majority of Tregs possess autoantigen-specific T cell receptors (TCR)^20,21^, a better understanding of Treg stability is essential to prevent undesirable therapeutic outcomes following transfusion of Tregs, including a loss of Foxp3 expression and the concomitant acquisition of effector functions.

Herein, we report that the serine/threonine kinase, p21-activated kinase 2 (Pak2), is essential for protecting Treg stability and preventing deviation into Th2-like effector cells. Pak2-deficient Tregs from Treg-specific Pak2-deficient mice failed to suppress T cell effector functions *in vitro* and *in vivo*. Rather than being suppressive, Pak2-deficient Tregs were poised to produce large amounts of Th2 cytokines following activation and showed an inability to control both Th1- and Th2-mediated inflammation in the periphery. Interestingly, absence of Pak2 in Tregs resulted in a loss of a characteristic Treg phenotype, shown by reduced expression of Foxp3, CD25, CTLA-4 and Nrp-1.All of these factors are thought to simultaneously heighten peripheral inflammation in Treg-specific Pak2-deficient mice, resulting in widespread multi-organ damage and lethality. Collectively, these results highlight Pak2 as a key signaling molecule in maintaining a suppressive Treg phenotype and protecting Tregs from diverting into Th2-like cells that are incapable of suppressing aberrant immune responses.

## Results

### Treg-specific loss of Pak2 results in a lethal, multi-organ autoimmune disease

Previously, our laboratory reported that *Cd4*-Cre mediated deletion of *Pak*2 in T cells resulted in a block in the development of thymic-derived and peripherally induced regulatory T cells (Tregs) coupled with the onset of spontaneous colitis, suggestive of a break in peripheral tolerance^22^. This block in development was, in part, due to an inability of Pak2-deficient Tregs to transduce the appropriate high-affinity TCR- and cytokine-driven signals that are a prerequisite for their development^22^. However, this Treg developmental defect in the absence of Pak2 prevented us from ascertaining whether the observed break in immune tolerance was a result of a loss of Treg numbers or a loss of Treg suppressive function. To circumvent this, we generated a mouse strain in which *Pak2* was deleted specifically in the Treg compartment, using a Cre-YFP recombinase expressed under the control of the Treg-specific *Foxp3* promoter^23^. As WT controls, we used *Pak2* ^+/+^;*Foxp3*-Cre mice throughout these studies.

**Figure 1:**
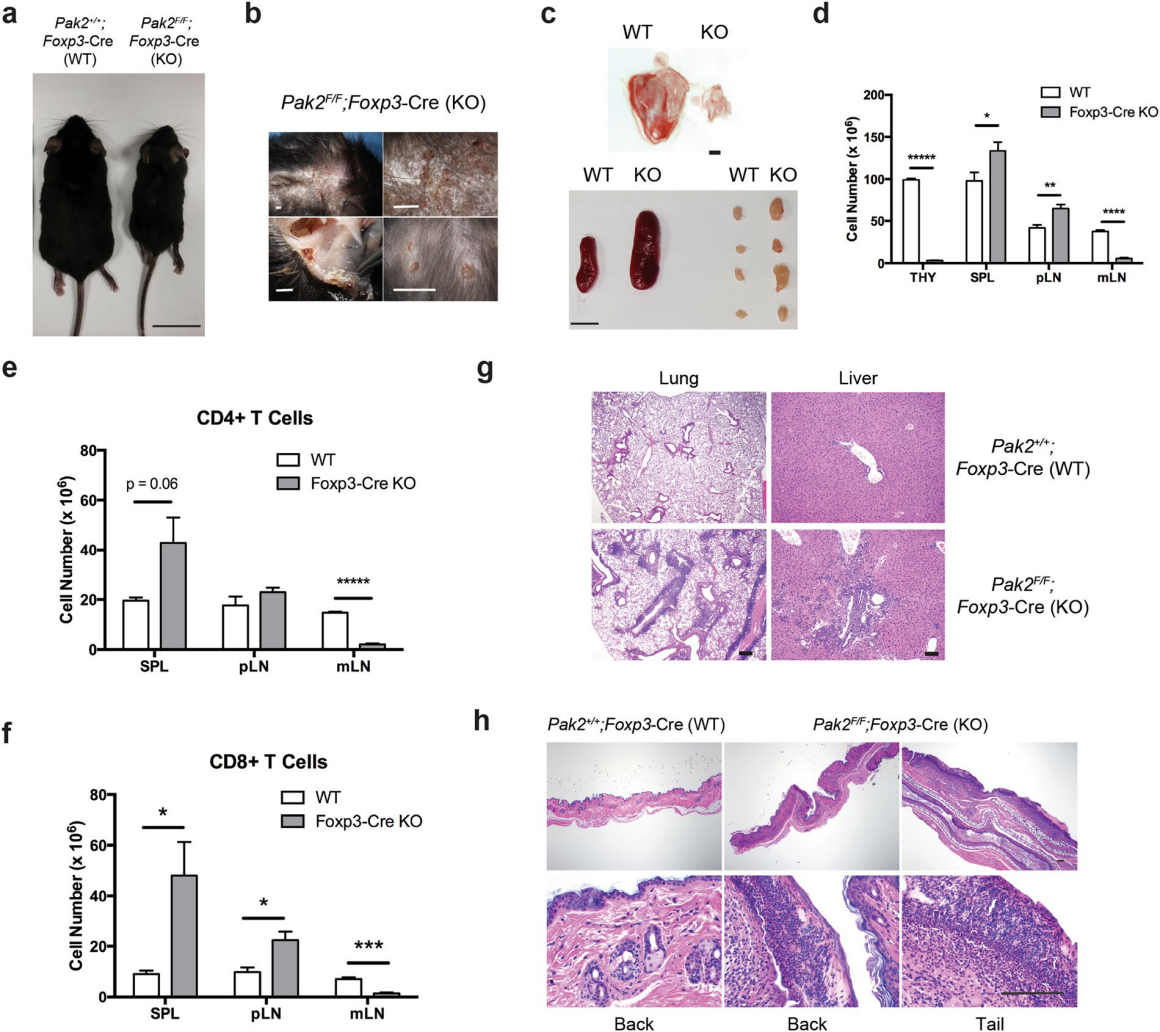
Treg-specific loss of Pak2 results in a lethal, multi-organ autoimmune disease. (**a**) Gross anatomical analysis illustrating runted growth (scale bar, 1 cm) in *Pak2* ^*F/F*^;*Foxp3*-Cre mice (*Foxp3*-Cre KO) compared to *Pak2* ^*F/F*^ (WT) mice. (**b**) Anatomical analysis of dermatitis lesions present on the body of *Foxp3*-Cre KO mice (scale bar, 1 mm). (**c**) Gross anatomical analysis of severe thymic atrophy (*left panel*), splenomegaly (*middle*) and lymphadenopathy (*right*) in *Foxp3*-Cre KO mice (scale bar, 1 mm for thymus, 1 cm for spleen and lymph nodes). (**d**) Total cell numbers from the thymus, spleen (SPL), peripheral lymph nodes (pLN) and mesenteric lymph nodes (mLN) of WT and *Foxp3*-Cre KO mice. (**e**) Total cell numbers of CD4+ T cells in the SPL, pLN and mLN of WT and *Foxp3*-Cre KO mice. (**f**) Total cell numbers of CD8+ T cells from the SPL, pLN and mLN of WT and *Foxp3*-Cre KO mice. (**g**) H&E staining of sections from the lung (*left panel*) and liver (*right panel*) of *Pak2* ^*F/F*^ (WT) and *Pak2* ^*F/F*^;*Foxp3*-Cre (*Foxp3*-Cre KO) mice. Scale bar, 100 μm (objectives of ×4, ×10, and ×40). (**h**) H&E staining of sections from the skin of the back and tail of WT (*left panel*) and *Foxp3*-Cre KO (*middle and right panel*) mice. Graphs within this figure show mean ± SE (n = 5). *0.01 <*p* < 0.05, **0.001 < *p* < 0.01, ***0.0001 < *p* 0.001, ****0.00001 <*p* < 0.0001, *****0.00001 <*p *< 0.000001 (unpaired two-tailed Student *t* test). Results are representative of at least three independent experiments.

Interestingly, *Foxp3*-Cre Pak2-deficient mice succumbed to death within 7–8 weeks after birth following the development of a more severe inflammatory disease compared to *Cd4*-Cre Pak2-deficient mice, evident by their runted size and the development of dermatitis, with multiple skin lesions apparent on the back, abdomen, tail and ears (Fig. 1a,b). Furthermore, *Foxp3*-Cre Pak2-deficient mice presented with splenomegaly, lymphadenopathy and severe thymic atrophy (Fig. 1c), suggestive of ongoing inflammation. Upon disease onset, the total cell numbers within the thymus and mesenteric lymph nodes (mLN) were significantly reduced, which was in contrast to a significant increase in total cell numbers in the spleen (SPL) and peripheral lymph nodes (pLN; pooled inguinal, brachial and axillary) (Fig. 1d). The number of total CD4+ and CD8+ T cells was increased in the spleen (Fig. 1e,f) but variable in the peripheral lymph nodes. Total cell numbers and CD4+ and CD8+ T cell numbers were markedly reduced in mesenteric lymph nodes (Fig. 1e,f).

Histological examination of multiple organs revealed significant inflammatory cell infiltration in the lungs, liver and skin of *Foxp3*-Cre Pak2-deficient mice (Fig. 1g,h). A combination of lymphocytes and plasmacytoid cells surrounded the airways in the lungs and the portal triad in the liver (Fig. 1g). As expected, the skin presented with multiple epidermal ulcers and the normal structure of the epidermis was replaced with necrotic debris and granulomatous tissue (Fig. 1h). Skin lesions showed deposition of fibroblasts and chronic inflammatory cells, including lymphocytes, plasma cells and granulocytes (Fig. 1g). These data suggested that mice with a Treg-specific deletion of Pak2 manifested with a severe and lethal multi-organ autoimmune disease, suggestive of a loss of Treg suppressive function.

### Treg-specific Pak2 deletion results in the activation of peripheral T cells

**Figure 2:**
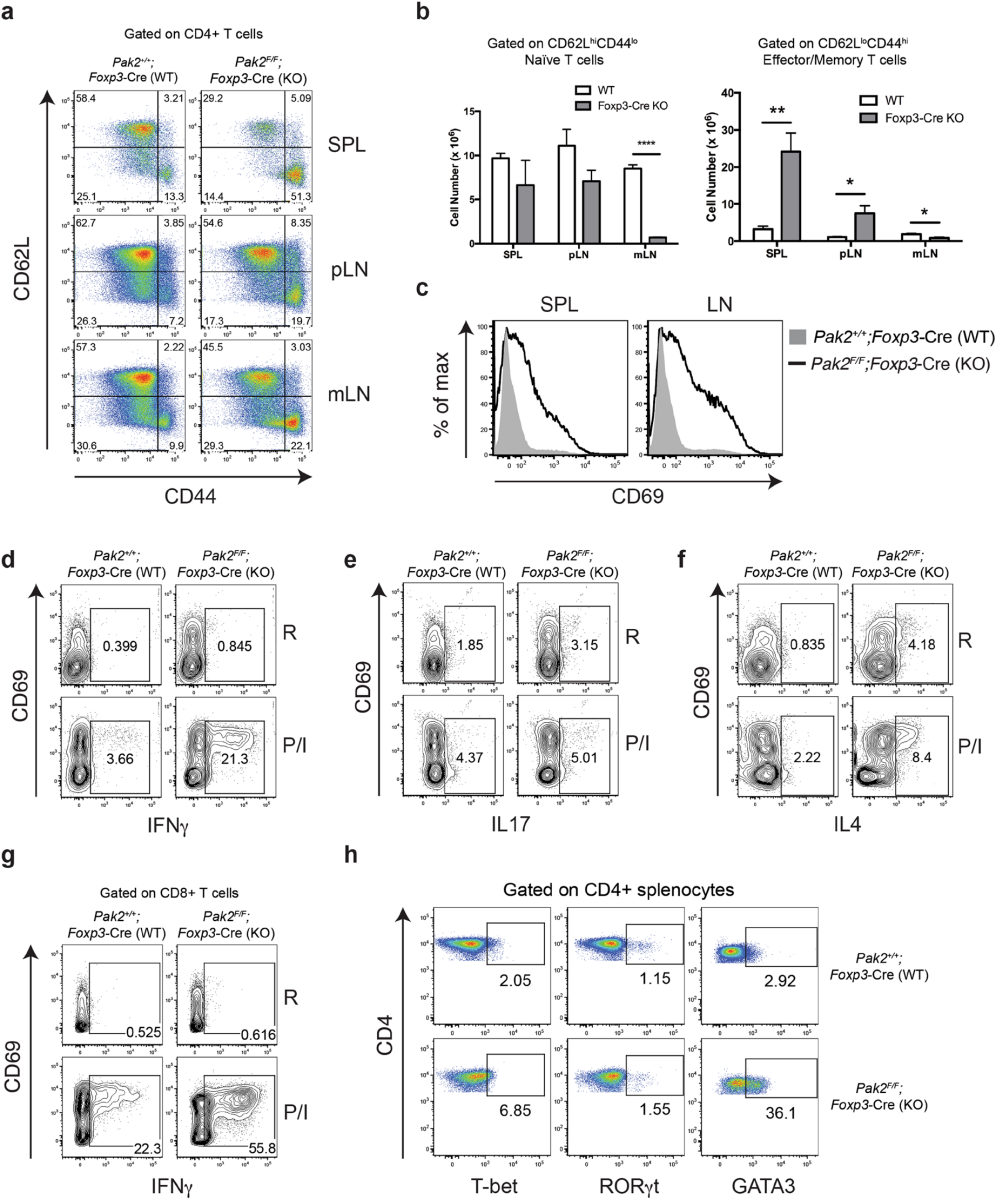
Pak2 deficiency in Tregs results in a significant increase in T cells with an activated memory phenotype. (**a**) Flow cytometric analysis of CD62L and CD44 expression within CD4+ T cells from the spleen (SPL), peripheral lymph nodes (pLN) and mesenteric lymph nodes (mLN) of *Pak2* ^*F/F*^ (WT) and *Pak2* ^*F/F*^;*Foxp3*-Cre (*Foxp3*-Cre KO) mice. (**b**) Total cell numbers of CD62L^hi^CD44^lo^ naïve T cells (*left panel*) and CD62L^lo^CD44^hi^ effector memory T cells (*right panel*) from WT and *Foxp3*-Cre KO mice. (**c**) Flow cytometric analysis of CD69 expression within CD4+ T cells from the SPL and total lymph nodes (LN) of WT and *Foxp3*-Cre KO mice. (**d–f**) Flow cytometric analysis of IFN-γ (**d**), IL-17 (**e**) and IL-4 (**f**) expression within CD4+ T cells from the spleen of *Pak2* ^*F/F*^ (WT) and *Pak2* ^*F/F*^;*Foxp3*-Cre (*Foxp3*-Cre KO) mice following stimulation of total splenocytes with PMA and ionomycin (P/I) for 4 hours (R: resting conditions in the absence of P/I). (**g**) Flow cytometric analysis of IFN-γ expression within CD8+ T cells from the spleen of WT and *Foxp3*-Cre KO mice following stimulation of total splenocytes with PMA and ionomycin for 4 hours. (**h**) Flow cytometric analysis of T-bet, RORγt and GATA3 expression within CD4+ T cells from the spleen of WT and *Foxp3*-Cre KO mice. Graphs within this figure show mean ± SE (n = 5). *0.01 < *p* < 0.05, **0.001 < *p* < 0.01, ****0.00001 < *p* < 0.0001 (unpaired two-tailed Student *t* test). Results are representative of at least three independent experiments.

Given the severe immunopathology, we investigated whether peripheral T cells displayed an activated phenotype in *Foxp3*-Cre Pak2-deficient mice. The absolute number of activated CD62L^lo^CD44^hi^ effector memory T cells in the spleen (SPL) and peripheral lymph nodes (pLN) was significantly increased, with little change in the total number of naïve CD62L^hi^CD44^lo^ CD4+ T cells in of *Foxp3*-Cre Pak2-deficient mice (Fig. 2a,b). Furthermore, CD69 expression was increased in CD4+ T cells from the spleen and pLN of *Foxp3*-Cre Pak2-deficient mice (Fig. 2c), supporting that T cells were activated in the periphery.

The presence of activated T cells in *Foxp3*-Cre Pak2-deficient mice suggested an inability of Pak2-deficient Tregs to appropriately regulate T cell responses. To determine whether Pak2-deficient Tregs were effective in controlling Th1, Th17 and/or Th2 effector responses, we measured the production of lineage-specific cytokines following stimulation of CD4+ T cells from WT (*Pak2* ^+/+^;*Foxp3*-Cre) and *Foxp3*-Cre Pak2-deficient mice (*Pak2* ^*F/F*^;*Foxp3*-Cre) with PMA and ionomycin. Of note, since we did not use skewing conditions for Th1, Th17 and Th2 differentiation, production of IFN-γ, IL-17 and IL-4 following stimulation in WT cells were increased, but not optimal. However, expression of IFN-γ and IL-4 in CD4+ T cells from *Foxp3*-Cre Pak2-deficient mice was markedly increased compared to splenic and lymph node CD4+ T cells, while expression of IL-17 remained unchanged (Fig. 2d,e,f). Furthermore, the proportion of CD4+ T cells expressing the Th1- and Th2-specific transcription factors, T-bet and GATA3, respectively, was markedly increased in *Foxp3*-Cre Pak2-deficient mice (Fig. 2h). Interestingly, the proportion of T cells expressing GATA3 was almost 10-fold higher in *Foxp3*-Cre Pak2-deficient mice compared to WT mice, while little change was observed in the proportion of CD4+ T cells expressing RORγt (Fig. 2h, *middle panel*). Additionally, the proportion of IFN-γ-producing CD8+ T cells was increased in the spleen and pLN of *Foxp3*-Cre Pak2-deficient mice (Fig. 2g). These data support that *Foxp3*-Cre Pak2-deficient Tregs were unable to control the expansion of Th1 and Th2 effector cells specifically.

**Figure 3:**
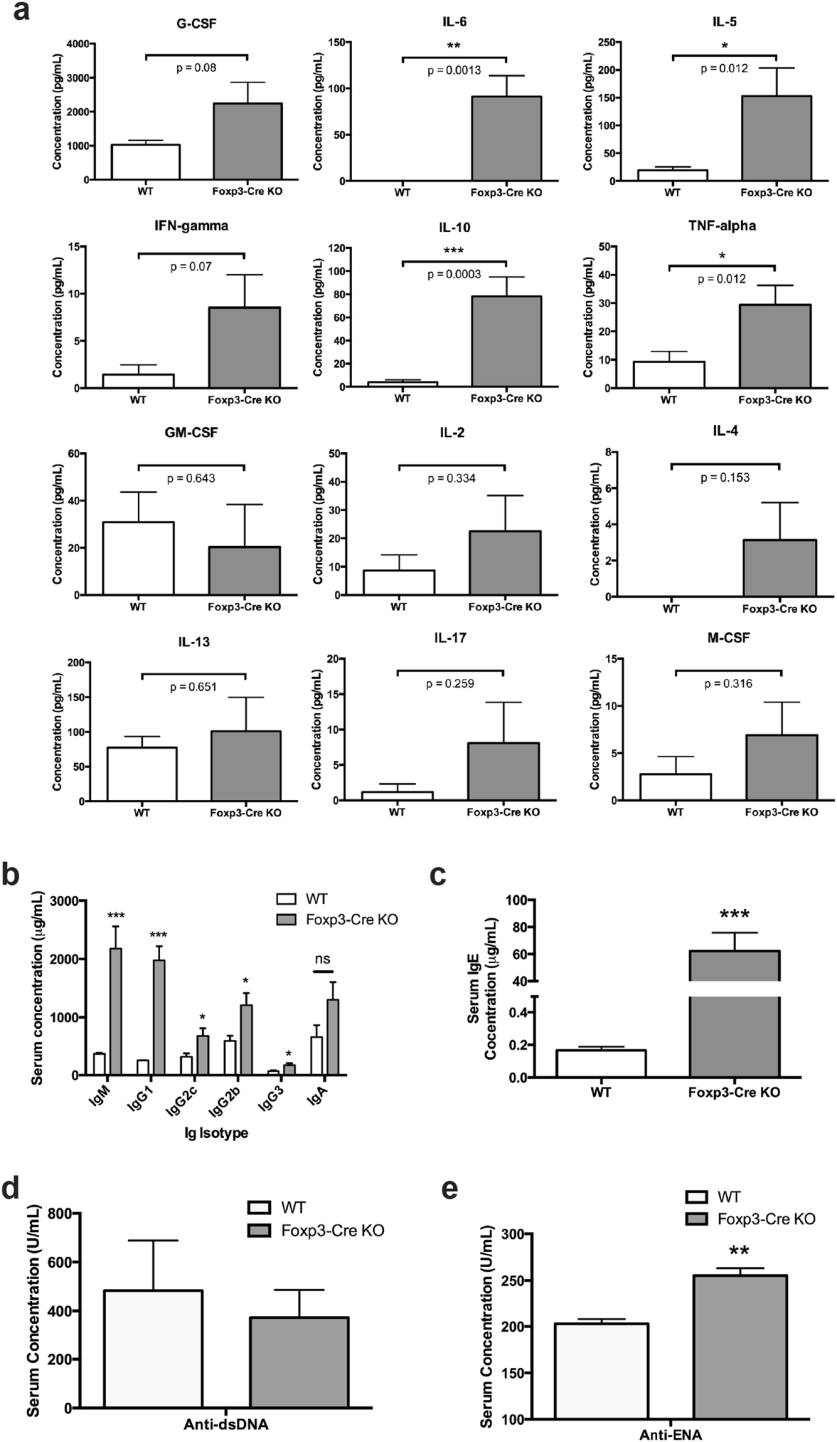
Loss of Pak2 in Tregs results in increased inflammatory markers in blood serum. (**a**) Concentration of G-CSF, IL-6, IL-5, IFN-γ, IL10, TNF-α, GM-CSF, IL-2, IL-4, IL-13, IL-17 and M-CSF in serum isolated from the blood of *Pak2* ^*F/F*^ (WT) and *Pak2* ^*F/F*^;*Foxp3*-Cre (*Foxp3*-Cre KO) mice. (**b**) Concentration of IgM, IgG1, IgG2c, IgG2b, IgG3 and IgA in serum isolated from the blood of WT and *Foxp3*-Cre KO mice. (**c**) Concentration of IgE in the serum isolated from the blood of WT and *Foxp3*-Cre KO mice. (**d**) Concentration of anti-double-stranded DNA (anti-dsDNA) and (**e**) anti-extranuclear antigen (anti-ENA) autoantibodies in the serum isolated from the blood of WT and *Foxp3*-Cre KO mice. Graphs in this figure show mean ± SE (n = 9 for (**a**); n = 3 for (**b–e**)). *0.01 < *p* < 0.05, **0.001 < *p* < 0.01, ***0.0001 < *p* 0.001 (unpaired two-tailed Student *t* test). Results are representative of at least three independent experiments performed in triplicate.

To show support for an increase in inflammatory responses in *Foxp3*-Cre Pak2-deficient mice, we next measured the serum levels of multiple cytokines. Although IL-6, TNF-α and G-CSF are implicated in a general inflammatory phenotype, these cytokines have also been implicated in driving Th2 cell differentiation^24–26^ and were markedly increased in the serum of *Foxp3*-Cre Pak2-deficient mice (Fig. 3a). Interestingly, the serum levels of IL-5 and IL-10, both produced by Th2 cells^27,28^, were significantly increased (Fig. 3a). Although levels did not reach statistical significance, IFN-γ was appreciably increased in the serum of *Foxp3*-Cre Pak2-deficient mice, further suggesting that, in addition to Th2 cells, Th1 cells may contribute to the observed inflammatory immunopathology. Of note, no changes were observed in other cytokines known to be produced by Th1 and Th17 cells, including IL-2, GM-CSF, M-CSF and IL-17A (Fig. 3a), possibly suggesting that a more Th2-dominant cytokine profile was present in the periphery of *Foxp3*-Cre Pak2-deficient mice. This was in line with the heightened proportion of GATA3-expressing CD4+ Th2 cells detected in *Foxp3*-Cre Pak2-deficient mice (Fig. 2h, *right panel*).

### Treg-specific Pak2 deletion results in significant B cell activation

In addition to T lymphocyte infiltration, tissue histology revealed an increase in the infiltration of plasma cells in multiple organs from *Foxp3*-Cre Pak2-deficient mice (Fig. 1g,h). Given the heightened activation of Th1 and Th2 effector cells, we hypothesized that activated B cells may contribute to disease.

To assess this, we used an antibody-specific ELISA to determine the serum concentrations of a variety of immunoglobulins in WT (*Pak2* ^+/+^;*Foxp3*-Cre) and *Foxp3*-Cre Pak2-deficient mice (*Pak2* ^*F/F*^;*Foxp3*-Cre). In striking contrast to WT mice, we observed a significant increase in the serum concentrations of IgM, IgG1, IgG2c, IgG2b and IgG3 in *Foxp3*-Cre Pak2-deficient mice, with a trend towards an increase in IgA serum levels (Fig. 3b). Furthermore, we observed an approximate 300-fold increase in serum IgE concentrations in *Foxp3*-Cre Pak2-deficient mice, relative to WT mice (Fig. 3c). While the serum levels of anti-dsDNA were comparable between WT and *Foxp3*-Cre Pak2-deficient mice, there was a slight increase in the serum levels of anti-ENA autoantibodies in the serum of *Foxp3*-Cre Pak2-deficient mice (Fig. 3c,d). Consequently, these findings supported that the immunopathology observed in *Foxp3*-Cre Pak2-deficient mice was, in part, caused by antibody-producing B cells that were aberrantly activated under inflammatory conditions.

### Tregs lose their characteristic phenotype in the absence of Pak2

The presence of activated T and B cells in *Foxp3*-Cre Pak2-deficient mice suggested a break in peripheral tolerance, likely caused by Tregs that lack suppressive function in the absence of Pak2. Consequently, we next investigated the phenotype of Pak2-deficient Tregs relative to WT counterparts.

**Figure 4:**
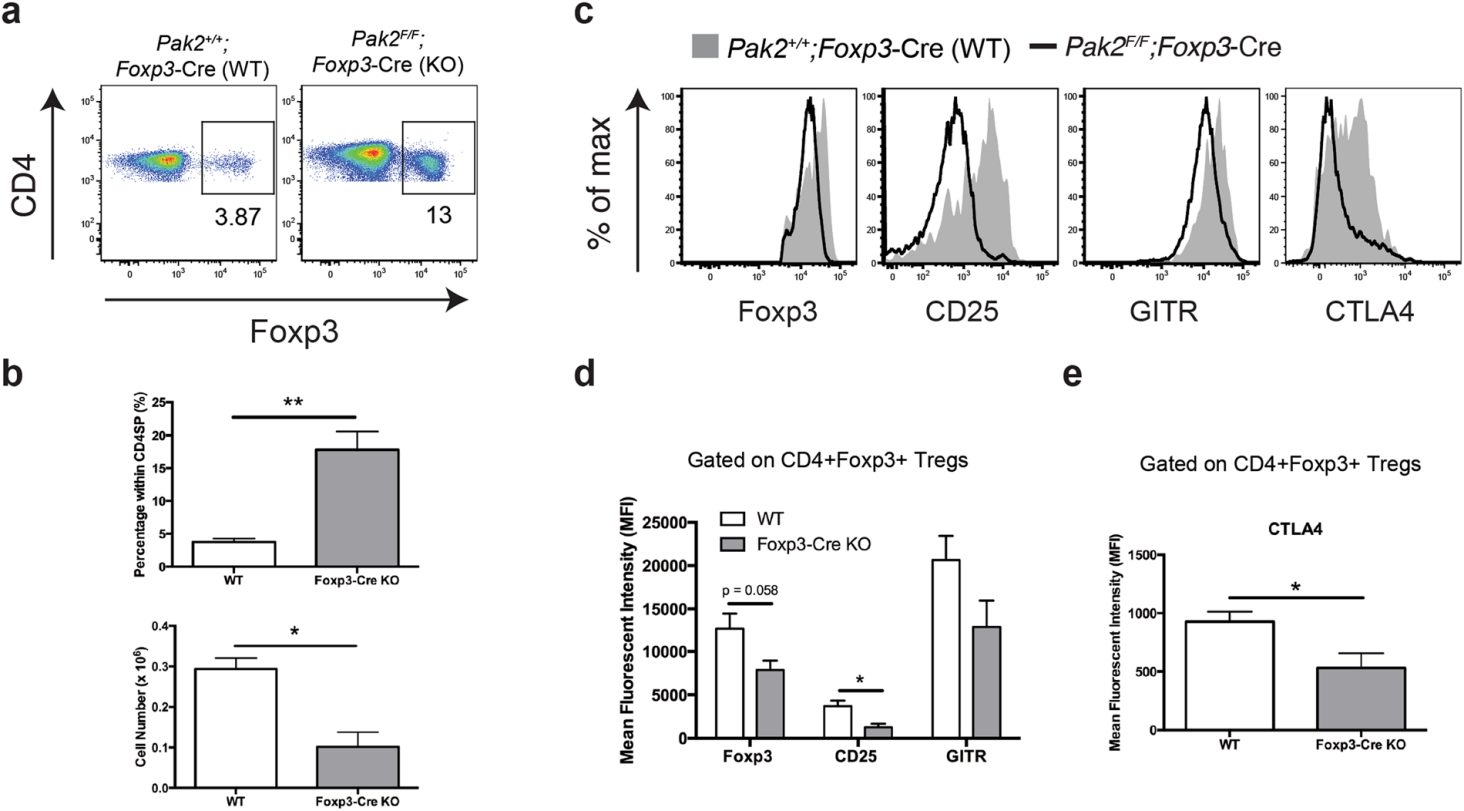
Pak2-deficient thymic Tregs lose their characteristic Treg phenotype. (**a**) Flow cytometric analysis of Foxp3 expression within CD4 single positive (CD4SP) thymocytes from *Pak2* ^*F/F*^ (WT) and *Pak2* ^*F/F*^;*Foxp3*-Cre (*Foxp3*-Cre KO) mice. (**b**) *Upper panel*: Percentage of CD4+Foxp3+ Tregs within total CD4SP thymocytes from the thymus of WT and *Foxp3*-Cre KO mice. *Bottom panel*: Total cell numbers of CD4+Foxp3+ Tregs from the thymus of WT and *Foxp3*-Cre KO mice. (**c**) Flow cytometric analysis of Foxp3, CD25, GITR and CTLA4 expression within Foxp3+ CD4SP thymocytes from WT and *Foxp3*-Cre KO mice. (**d**) Mean fluorescent intensity (MFI) of Foxp3, CD25 and GITR within CD4+Foxp3+ Tregs from the thymus of WT and *Foxp3*-Cre KO mice. (**e**) MFI of CTLA4 within CD4+Foxp3+ Tregs from the thymus of WT and *Foxp3*-Cre KO mice. Graphs within this figure show mean ± SE (n = 5). *0.01 < *p* < 0.05, **0.001 < *p* < 0.01 (unpaired two-tailed Student *t* test). Results are representative of at least three independent experiments.

The absolute number of thymic CD4+Foxp3+ Tregs (tTregs) in *Foxp3*-Cre Pak2-deficient mice was significantly reduced, due to severe thymic atrophy (Fig. 4a,b), but the proportion of tTregs within CD4 single positive thymocytes was increased. Importantly, Pak2-deficient tTregs exhibited a significant reduction in their expression of CD25 and CTLA-4, with little change in GITR expression (Fig. 4c,d,e). Furthermore, there was an appreciable reduction in Foxp3 expression levels in Pak2-deficient tTregs, although the difference in Foxp3 expression did not reach statistical significance (Fig. 4b,d). These data suggested that, in the absence of Pak2, tTregs lost their characteristic Treg phenotype, as evident by a significant decrease in expression of CD25 and CTLA4 and a reduction in Foxp3 expression.

**Figure 5:**
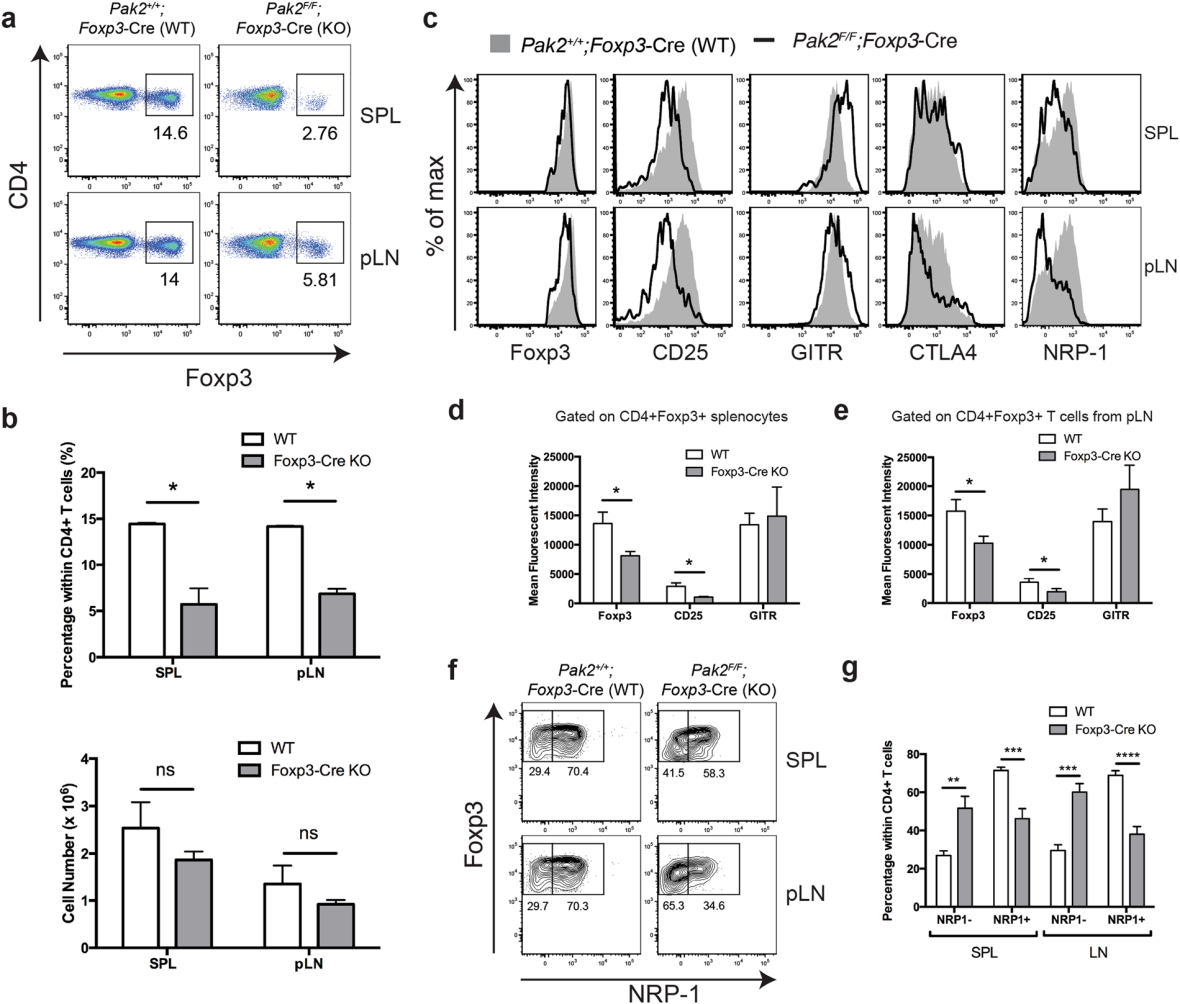
Pak2-deficient Tregs maintain an aberrant phenotype in the periphery. (**a**) Flow cytometric analysis of Foxp3 expression within CD4+ T cells from the spleen (SPL) (*top panels*) and peripheral lymph nodes (pLN) (*bottom panels*) of *Pak2* ^*F/F*^ (WT) and *Pak2* ^*F/F*^;*Foxp3*-Cre (*Foxp3*-Cre KO) mice. (**b**) *Upper panel*: Percentage of CD4+Foxp3+ Tregs within total CD4+ T cells from the SPL and pLN of WT and *Foxp3*-Cre KO mice. *Bottom panel*: Total cell numbers of CD4+Foxp3+ Tregs from the SPL and pLN of WT and *Foxp3*-Cre KO mice. (**c**) Flow cytometric analysis of Foxp3, CD25, GITR, CTLA4 and NRP-1 expression within Foxp3+ CD4+ T cells from the SPL and pLN of WT and *Foxp3*-Cre KO mice. (**d**) Mean fluorescent intensity (MFI) of Foxp3, CD25 and GITR within CD4+Foxp3+ Tregs from the spleen of WT and *Foxp3*-Cre KO mice. (**e**) MFI of Foxp3, CD25 and GITR within CD4+Foxp3+ Tregs from the pLN of WT and *Foxp3*-Cre KO mice. (**f**) Flow cytometric analysis of NRP-1 expression within CD4+Foxp3+ Tregs from the SPL and pLN of WT and *Foxp3*-Cre KO mice. (**G**) Percentage of NRP1− and NRP1+ Tregs within CD4+ T cells from the SPL and pLN of WT and *Foxp3*-Cre KO mice. Graphs within this figure show mean ± SE (n = 5). *0.01 < *p* < 0.05, **0.001 < *p* < 0.01, ***0.0001 < *p* 0.001, ****0.00001 < *p* < 0.0001, *****0.00001 < *p* < 0.000001 (unpaired two-tailed Student *t* test). Results are representative of at least three independent experiments.

Next, we investigated whether this thymic Treg phenotype translated to peripheral Tregs from *Foxp3*-Cre Pak2-deficient mice. In contrast to tTregs, the proportion of CD4+Foxp3+ Tregs from the spleen and pLN of *Foxp3*-Cre Pak2-deficient mice was significantly decreased, but the absolute number of peripheral Tregs was only marginally reduced in *Foxp3*-Cre Pak2-deficient mice (Fig. 5a,b). These results suggest that the reduced proportion of Tregs in *Foxp3*-Cre Pak2-deficient mice was likely due to increased effector CD4 and CD8 T cells (as shown in Fig. 2b). Similar to tTregs, we found a significant reduction in the expression of multiple Treg-associated surface molecules in the absence of Pak2, including Foxp3, CD25 and CTLA-4, with no appreciable change in GITR expression (Fig. 5c,d,e), suggesting an inability for Pak2-deficient Tregs to restore expression of important molecules that regulate their suppressive function.

Interestingly, we also observed a significant reduction in the expression of neuropilin-1 (NRP-1) on the surface of Pak2-deficient Tregs relative to WT Tregs (Fig. 5c). When gating on Foxp3 and NRP-1, WT peripheral Tregs could be differentiated into two distinct populations: NRP-1^lo^ and NRP-1^hi^, with the majority of WT Tregs expressing higher levels of NRP-1 (Fig. 5f). This was in support of literature stating that the majority of peripheral Tregs are thought to be derived from the tTreg pool that express high levels of NRP-1^29^. In the absence of Pak2, peripheral Tregs showed a significant reduction in the NRP-1^hi^ population, coupled with a significant increase in the frequency of NRP-1^lo^ Tregs (Fig. 5f,g). This suggested that Pak2 may either be responsible for maintaining NRP-1 expression in Tregs or, alternatively, may be important for maintaining the homeostasis of the NRP-1^hi^ tTreg population. Irrespective, these data highlighted that Pak2 is essential for maintaining a Treg repertoire that expresses multiple important functional Treg-associated proteins.

In our hands, we have observed similar inflammatory phenotypes between male (*Pak2* ^*F/F*^;*Foxp3*-Cre+) and female (*Pak2* ^*F/F*^;*Foxp3*-Cre+/+) mice that possess a homozygous deletion of *Pak2*, specifically within Tregs. This has been observed at both a gross anatomical level (*data not shown*) and a molecular level when measuring the expression levels of several Treg-associated markers by flow cytometry (*data not shown*).

To determine whether these changes in Treg phenotype were T cell-intrinsic, we also investigated the expression levels of several Treg-associated markers within Tregs derived from heterozygous female (*Pak2* ^*F/F*^;*Foxp3*-Cre+/−) mice in which approximately 50% of the Tregs possess a deletion of Pak2 while the remaining 50% of Tregs express Pak2 at wild-type levels, mimicking a mixed chimeric mouse model. This was also an ideal system to investigate whether the severe inflammation within homozygous *Pak2* ^*F/F*^;*Foxp3*-Cre+/+ mice was the underlying cause for the loss of a characteristic Treg phenotype.

Interestingly, we found that the reduction in Foxp3 expression within homozygous Pak2-deficient mice (*Pak2* ^*F/F*^;*Foxp3*-Cre+/+; Supplementary Fig. 1a,b) was likely caused by the severe inflammatory milieu within these mice. In support of this, Foxp3 expression in Pak2-deficient Tregs was similar to that observed in WT Tregs from heterozygous female *Pak2* ^*F/F*^;*Foxp3*-Cre+/− mice, suggesting that the presence of WT Tregs within the same host could restore Foxp3 expression in Pak2-deficient Tregs. Thus, the loss in Foxp3 expression in homozygous *Pak2* ^*F/F*^;*Foxp3*-Cre+/+ mice was not cell-intrinsic. However, changes in other Treg-associated markers, such as CD25 and GITR expression, were significantly altered in Pak2-deficient Tregs relative to WT Tregs from heterozygous female *Pak2* ^*F/F*^;*Foxp3*-Cre+/− mice (Supplementary Fig. 1a). This suggested that Pak2 might still perform a cell-intrinsic role in regulating the expression of certain important Treg-associated molecules.

### Loss of Pak2 in Tregs results in a loss of Treg suppressive function, *in vitro* and *in vivo*

The onset of the severe immunopathology observed in *Foxp3*-Cre Pak2-deficient mice, coupled with the attenuated expression of functional Treg-associated molecules, suggested that Pak2-deficient Tregs had lost their ability to suppress peripheral inflammation. We thus hypothesized that Treg function might be impaired either by the loss of an appropriate effector Treg population in the periphery or, alternatively, an inability of Pak2-deficient Tregs to directly suppress the proliferation and/or effector functions of conventional T cells (T_conv_) in the periphery.

**Figure 6:**
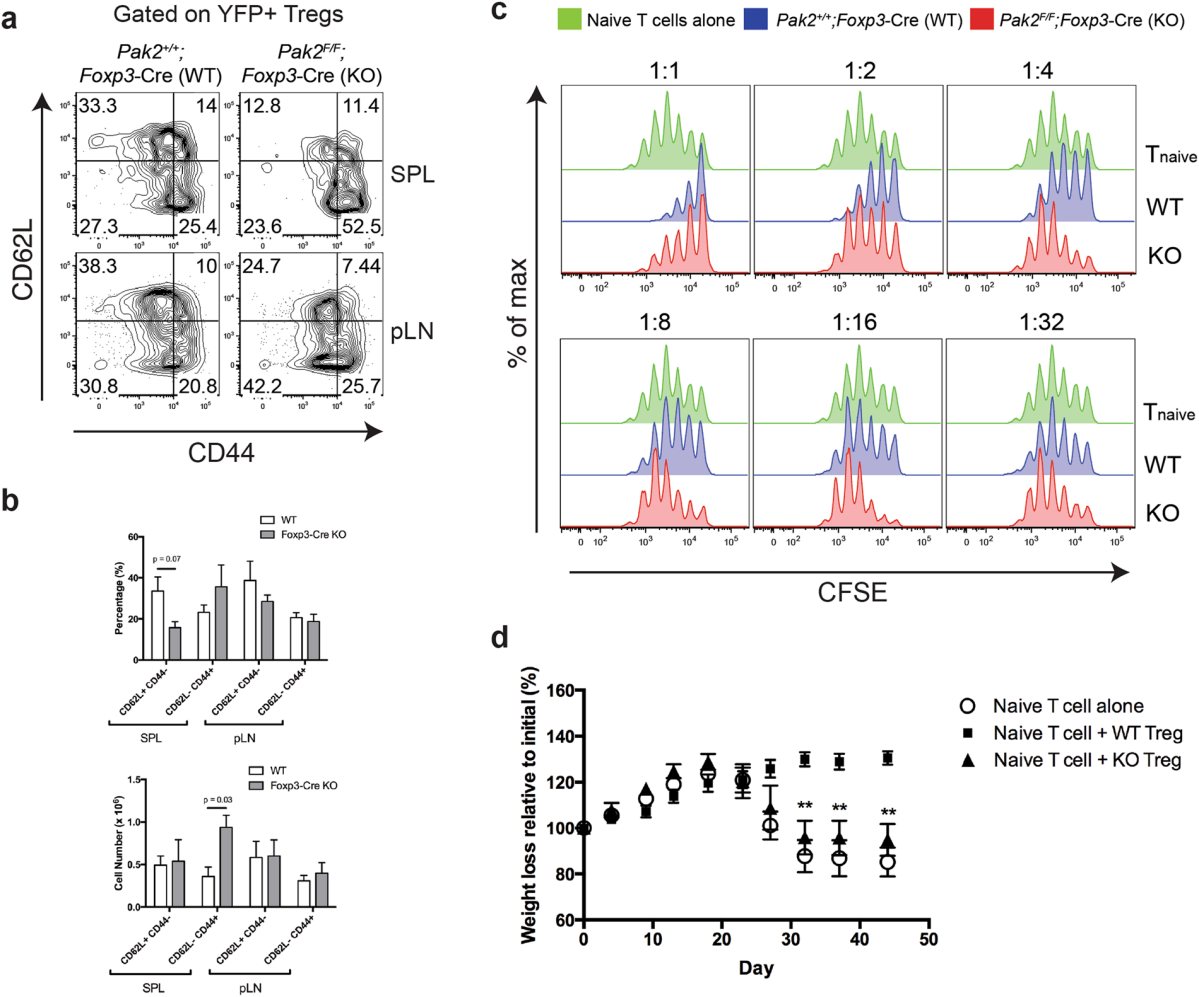
Loss of Pak2 in Tregs results in impaired Treg suppressive function *in vitro* and *in vivo*. (**a**) Flow cytometric analysis of CD62L and CD44 expression within YFP+ Tregs from *Pak2* ^*F/F*^ (WT) and *Pak2* ^*F/F*^;*Foxp3*-Cre (KO) mice. (**b**) The percentage (*upper panel*) and absolute number (*lower panel*) of naïve (CD62L+CD44−) and effector (CD62L−CD44+) Tregs from the spleen and pLN of WT and KO mice. Results are representative of at least 3 independent experiments. (**c**) Flow cytometric analysis of CFSE dilution following incubation of CFSE-labeled naïve conventional T cells (CD4+CD25−) with YFP+ WT (red histogram) or KO (blue histogram) Tregs. Green histograms represent the proliferation of CFSE-labeled conventional T cells without addition of Tregs. Cells were stimulated for 72 hours using a combination of irradiated splenocytes and anti-CD3 (1 μg/mL). Ratios indicate the ratio of CFSE-labeled conventional T cells to unlabeled Tregs. Results are representative of at least 2 independent experiments. (**d**) Percentage weight loss relative to initial recorded weight of Rag1^−/−^ mice injected with either CD4+CD25−CD45RB^hi^ naïve conventional T cells alone (Control, open circles) or naïve conventional T cells in combination with YFP+ WT Tregs (filled squares) or YFP+ Pak2-deficient Tregs (filled triangles). Mice were weighed every 4 days until the endpoint at 45 days. Graphs within this figure show mean ± SE (n = 5 per group). **0.001 < *p* < 0.01 (unpaired two-tailed Student *t* test). Results are representative of at least 3 independent experiments.

To determine whether *Foxp3*-Cre Pak2-deficient mice possessed an adequate number of effector Tregs, we isolated cells from the spleen and pLN and compared the proportion and number of naïve (CD62L^hi^CD44^lo^) versus effector memory (CD62L^lo^CD44^hi^) Foxp3+ Tregs in WT (*Pak2* ^+/+^;*Foxp3*-Cre) and *Foxp3*-Cre Pak2-deficient mice (*Pak2* ^*F/F*^;*Foxp3*-Cre). While the proportion of naïve CD62L+CD44− Tregs was reduced in the spleen of *Foxp3*-Cre Pak2-deficient mice, the absolute number of naïve Tregs was not statistically different (Fig. 6a,b). There was a trend towards an increase in the absolute number of effector CD62L−CD44+ Tregs in the spleen of *Foxp3*-Cre Pak2-deficient mice relative to WT littermates, although this did not reach statistical significance (Fig. 6a,b). No apparent differences were observed in the naïve versus effector Treg populations in the pLN of WT and *Foxp3*-Cre Pak2-deficient mice (Fig. 6a,b). Collectively, these data highlighted that the immunopathology was likely not caused by a loss of the effector Foxp3+ Treg population in *Foxp3*-Cre Pak2-deficient mice.

Next, we performed *in vitro* Treg functional assays to monitor the ability of Pak2-deficient Tregs to suppress the proliferation and effector functions of T_conv_ cells in response to anti-CD3 + anti-CD28 stimulation. In brief, CD25+YFP+ Tregs sorted from WT and *Foxp3*-Cre Pak2-deficient mice were co-cultured with freshly isolated CFSE-labeled CD4+CD25− naïve T_conv_ cells in combination with irradiated splenocytes from Rag1-deficient mice. After 72 hours, CFSE dilution was measured as a readout of T_conv_ cell proliferation. In the absence of Tregs, T_conv_ cells robustly divided, as evident by significant CFSE dilution, with approximately 5 cell divisions observed (Fig. 6c; *green histogram*). In the presence of a 1:1 ratio of WT Tregs, T_conv_ cell proliferation was markedly reduced, with only 3 cell divisions observed (Fig. 6c, *blue histogram*). T_conv_ cell proliferation gradually increased in a dose-dependent manner as the proportion of WT Tregs was reduced (Fig. 6c; *blue histogram*). In contrast, Pak2-deficient Tregs were inferior in suppressing the proliferation of T_conv_ cells when they were administered at a 1:2 ratio (Treg: T_conv_) or 1:4 ratio *in vitro* (Fig. 6c). Adding equal numbers of Pak2-deficient Tregs together with T_conv_ cells suppressed the proliferation of T_conv_ cells to a similar extent compared to WT Tregs, suggesting that the defects by Pak2-deficient Tregs could be overcome by administering more Tregs.

To confirm our findings *in vivo*, we used a T cell transfer model of colitis to assess the ability of Pak2-deficient Tregs to suppress the development of wasting disease in syngeneic T cell-deficient hosts^30,31^. In brief, Tregs were sorted from WT and *Foxp3*-Cre Pak2-deficient mice and transferred in combination with sorted naïve T_conv_ cells (CD4+CD25−CD45RB^hi^) into Rag1-deficient mice. As a positive control, T_conv_ cells alone were transferred into Rag1-deficient hosts. According to this model, signs of wasting disease should develop within 3–6 weeks following T cell transfer, mostly evident by significant weight loss due to an inability to control the inflammatory response to dietary and gut microbial antigens^30^.

Mice were monitored for changes in general appearance, including hunching and ruffled fur, as well as total body weight loss. While mice that received WT Tregs continued to gain weight throughout the experimental time course, mice that had received T_conv_ cells alone lost a significant amount of body weight between 30 and 45 days post-T cell transfer (Fig. 6d). Importantly, mice that had received T_conv_ cells in combination with *Foxp3*-Cre Pak2-deficient Tregs also lost a significant amount of body weight (Fig. 6d), suggesting that Pak2-deficient Tregs were unable to suppress the onset of wasting disease and, consequently, lacked appropriate Treg suppressive function.

Because we saw expression of Foxp3 was decreased in *Foxp3*-Cre Pak2-deficient Tregs (Fig. 5c–e), we asked whether this loss in Treg suppressive function in the *in vivo* Treg suppression assay was a result of losing Foxp3 expression upon homeostatic expansion within these hosts. To test this, YFP+ WT (*Pak2* ^+/+^;*Foxp3*-Cre+/+) and Pak2-deficient (*Pak2* ^*F/F*^;*Foxp3*-Cre+/+) Tregs were sorted and transferred into Rag1-deficient hosts and allowed to undergo homeostatic expansion for approximately 4 weeks. Following this, cells were isolated from the spleens and peripheral lymph nodes of mice that received either WT or Pak2-deficient Tregs and several markers were analyzed by flow cytometry, including Foxp3, CD25 and GITR (Supplementary Fig. 2a–d).

We noted that the proportion of Foxp3+ Tregs in mice that received Pak2-deficient Tregs was significantly lower than mice that had received WT Tregs (Supplementary Fig. 2a,b), while the proportion of CD4+Foxp3- cells remained similar (Supplementary Fig. 2a,b). This could indicate that Pak2-deficient Tregs transferred into Rag1-deficient hosts might expand, but lose Foxp3 expression upon expansion. Alternatively, Pak2-deficient Tregs may exhibit impaired survival when transferred into immunodeficient hosts. While this data cannot definitively differentiate between these two hypotheses, these results suggest that Pak2 may control the stability of Foxp3 expression or maintenance or survival of Foxp3+ Treg cells.

Interestingly, of those Pak2-deficient Tregs that were present within Rag1-deficient hosts, there was a statistically significant reduction in GITR, CTLA-4 (for splenic Tregs) and Foxp3 expression relative to WT Tregs (Supplementary Fig. 2c,d), suggesting that Pak2 is, indeed, important for maintaining a proper Treg phenotype, under homeostatic proliferation conditions.

### Loss of Pak2 alters the Treg genetic signature and skews cells towards a Th2 profile

Severe defects in Treg function and expression of Treg-signature proteins in the absence of Pak2 suggested that Pak2 is critical for maintaining Treg function and stability. To address whether Pak2 maintains a functional Treg genetic signature, we performed a cDNA microarray of WT (*Pak2* ^+/+^;*Foxp3*-Cre) and *Foxp3*-Cre Pak2-deficient (*Pak2* ^*F/F*^;*Foxp3*-Cre) Tregs to determine the gene expression profile regulated by Pak2. In total, Pak2 differentially regulated approximately 600 genes (*data not shown*). Ingenuity Pathway Analysis (IPA) revealed that the majority of the top canonical pathways that were altered in Pak2-deficient Tregs were associated with immune signaling pathways (Supplementary Fig. 3a,b). Additionally, immunological diseases and the inflammatory response were amongst the top diseases and biological functions that were altered in Pak2-deficient Tregs (Supplementary Fig. 3a). Interestingly, the sub-categories that were enriched within the immunological disease category included many autoimmune diseases including rheumatoid arthritis and Th2-allergic inflammatory diseases, including hypersensitive reaction, atopic dermatitis and allergic inflammation (*data not shown*).

**Figure 7:**
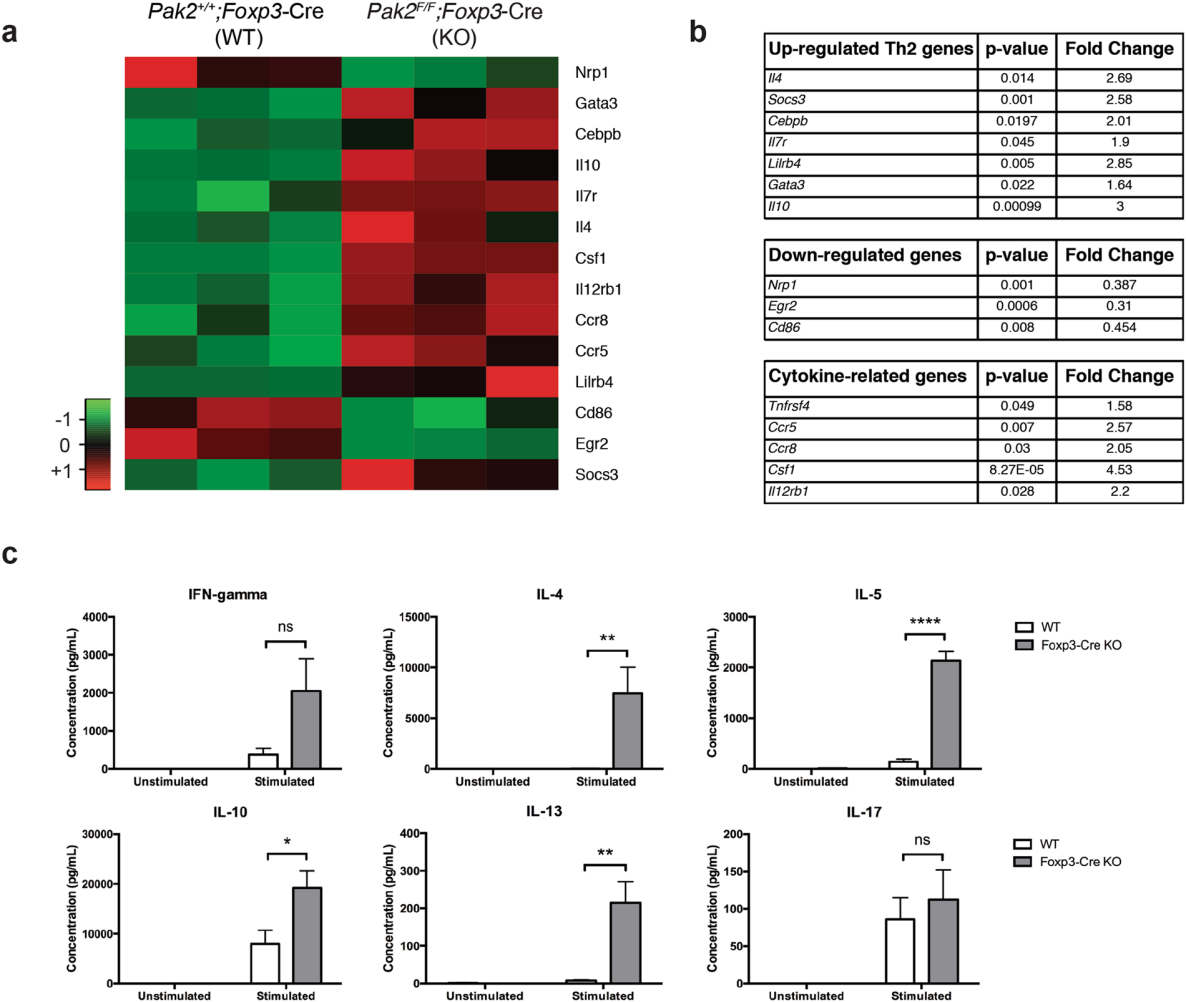
Pak2-deficient Tregs acquire properties of Th2 cells. Heat map illustrating the differential expression profile of specific genes of interest within Tregs derived from *Pak2* ^*F/F*^ (WT) and *Pak2* ^*F/F*^;*Foxp3*-Cre (KO) mice using a comparative cDNA microarray. The color key indicates the relative Z-score as a measure of changes in gene expression. (**b**) The *p*-value and relative fold change of several Th2-associated genes (*upper panel*), TCR-associated genes (*middle panel*) and cytokine/chemokine-related genes (*lower panel*) in KO Tregs relative to WT Tregs from the cDNA microarray. These data are representative of three independent biological replicates. (**c**) Concentration of IFN-γ, IL-4, IL-5, IL-13, IL10 and IL-17 from culture supernatants following TCR stimulation of sorted YFP+ WT and Pak2-deficient Tregs for 72 hours. *Unstimulated*: YFP+ Tregs were cultured in complete RPMI-1640 media supplemented with IL-2 (50 U/ml). *Stimulated*: YFP+ Tregs were cultured in complete RPMI-1640 media with IL-2 (50 U/ml) and stimulated with plate bound anti-CD3 (10 μg/ml) and soluble anti-CD28 (2 μg/ml). Graphs within this figure show mean ± SE (n = 3). *0.01 < *p* < 0.05, **0.001 < *p* < 0.01, ****0.00001 < *p* < 0.0001 (two-way ANOVA).

A more in-depth analysis of specific genes that were differentially regulated by Pak2 revealed striking enrichment of genes that are involved in Th2 effector differentiation and function in Pak2-deficient Tregs. A significant upregulation of *Il4*, *Il7r*, *Gata3* and *Il10* was found in Pak2-deficient Tregs (Fig. 7b,c), suggesting that Pak2-deficient Tregs might adopt a phenotype that more resembled Th2 cells. Similarly, genes that are involved in cytokine- and chemokine-related signaling were also enriched in Pak2-deficient Tregs (Fig. 7b,c, and Supplementary Fig. 3c,d). On the contrary, *Foxp3* mRNA expression was slightly reduced in the absence of Pak2. Moreover, TCR-regulated genes such as *Nrp1* and *Egr2* were reduced (Fig. 7b,c, Supplementary Fig. 3c), suggesting Pak2 contributes to TCR signaling in Tregs similar to conventional T cells^22,48^.

Given the overrepresentation of Th2-associated genes in Pak2-deficient Tregs, we determined whether, in the absence of Pak2, Tregs acquired Th2 effector function. To test this directly, we sorted WT (*Pak2* ^+/+^;*Foxp3*-Cre) and Pak2-deficient Tregs (*Pak2* ^*F/F*^;*Foxp3*-Cre) using the YFP marker that is present in the *Foxp3*-Cre transgene cassette. Foxp3+ Tregs were either left unstimulated or stimulated with plate-bound anti-CD3 and soluble anti-CD28, in the presence of IL-2, and secreted cytokine levels within culture supernatants were determined 72 hours post-stimulation. Strikingly, we observed significant increases in the production of the Th2-associated cytokines, IL-4, IL-5, IL-10 and IL-13, in the supernatants of stimulated Pak2-deficient Tregs (Fig. 7c). In contrast, no significant differences were observed in the production of Th1 and Th17 cytokines, IFN-γ and IL-17, respectively (Fig. 7c), suggesting that Pak2-deficient Tregs specifically acquired the ability to produce Th2 cytokines. Interestingly, *Gata3* mRNA expression was slightly increased in *Foxp3*-Cre Pak2-deficient Tregs (1.64 fold increase, *p*=0.022), although we cannot rule out the fact that there might be other factors regulating the significant expression of Th2 cytokines generated by these cells.

In sum, these data suggest that, in the absence of Pak2, Tregs lose suppressive function as well as important Treg-associated molecules, failing to control severe inflammation. Remaining Pak2-deficient Tregs start to lose expression of Foxp3 and begin to express genes that are typically suppressed within the Treg lineage and acquire the genetic signature and effector functions of Th2 cells. Based on these results, we propose that Pak2 safeguards Tregs from losing their suppressive phenotype and stability and protects them from diverting into effector T cells under inflammatory conditions.

## Discussion

With an established role in maintaining peripheral immune homeostasis, significant research efforts have been dedicated to understanding the signaling mechanisms that drive the development and function of Foxp3+ Tregs^1,2,32,33^. Previous work from our laboratory provided the first evidence to support a role for Pak2 in transducing critical high-affinity TCR and cytokine signals that are a prerequisite for the proper generation of thymic-derived Tregs^22^. We extended this knowledge in the current report to show that Pak2 was additionally required for proper Treg suppressive function, as well as the maintenance of a proper Treg phenotype with characteristic genetic and suppressive signatures.

The first evidence to suggest a role for Pak2 in Treg function followed the observation that *Pak2* ^*F/F*^;*Foxp3*-Cre mice developed a severe and lethal multi-organ lymphoproliferative disorder, similar to that observed in *scurfy* mice possessing a mutation in the *Foxp3* allele^34,35^. In the absence of Pak2, Tregs showed a dramatic reduction in expression of several key functional molecules, including CD25, CTLA-4 and NRP-1, all of which have been implicated in regulating the suppressive capacity of Tregs^36–38^. Interestingly, these molecules have also been described as predicted targets of Foxp3^36,39,40^, expression of which was reduced to 60% of WT in peripheral Pak2-deficient Tregs. With a combined reduction in Foxp3, CD25, CTLA-4 and NRP-1 expression in peripheral Pak2-deficient Tregs, we predicted that Treg suppressive function would be significantly impaired in *Pak2* ^*F/F*^;*Foxp3*-Cre mice. Indeed, while WT Tregs were shown to effectively suppress conventional T cell (T_conv_) proliferation *in vitro* and *in vivo*, Tregs isolated from *Pak2* ^*F/F*^;*Foxp3*-Cre mice showed significant disadvantages in the same assays, suggesting that the suppressive capacity of Pak2-deficient Tregs was impaired.

As a means to better understand the global effect of Pak2 deletion on Treg phenotype and function, we compared the genetic signatures of WT and Pak2-deficient Tregs. In-depth analysis revealed an enrichment of Th2-associated genes in Pak2-deficient Tregs, including enhanced expression of *Gata3* and *Il4*. Consistent with these data, IL4 signaling was identified as a top canonical pathway affected by the absence of Pak2 in Tregs. GATA3 has been considered the master regulator of Th2 differentiation through an ability to establish a Th2 genetic signature, including expression of IL-4^41^. Autocrine IL4 signaling is able to augment GATA3 expression through a positive feedback loop, while also regulating expression of multiple Th2-associated genes, including *Socs3*, *Il7r*, *Cebpb*, *Tnfsf14* and *Il10*, in a STAT6-dependent manner^42^. Strikingly, all of these genes were overrepresented in Pak2-deficient Tregs, suggesting that, in the absence of Pak2, Tregs lose their characteristic Treg genetic signature and acquire expression of genes that are typically restricted to Th2 cells.

Although controversial, several studies have shown the ability of Tregs to acquire Th1 and Th17 cytokine producing capabilities depending on the surrounding cytokine milieu^3,19^. These modified cells have been termed ‘ex-Tregs’ as a consequence of losing their characteristic suppressive activity and adopting a more pro-inflammatory phenotype^3^. While expression of GATA3 has been identified in Tregs^43^, there is limited data available describing the molecular signals protecting Tregs from acquiring a Th2 ex-Treg phenotype. A Treg-specific deletion of the E3 ubiquitin ligase, Itch, resulted in a widespread autoimmune disorder that was shown to be due to acquisition of Th2-cytokine production by Foxp3+ Tregs^44^. The authors proposed that these Th2-like Tregs were then capable of augmenting the conversion of naïve conventional T cells to Th2 cells, promoting allergic inflammation in these mice^44^. Additionally, several studies have shown that Tregs with attenuated Foxp3 expression preferentially convert to Th2 cells^18^, although the molecular mechanisms that cause this Treg instability remain ill defined. Our findings support that Pak2-deficient Tregs exhibit attenuated Foxp3 expression, coupled with acquisition of Th2 effector functions, suggesting that Pak2 may be an essential signaling molecule in regulating Treg stability and the balance between Treg and Th2 conversion.

Several possibilities might explain how a Treg-specific loss of Pak2 could skew Foxp3+ Tregs towards a phenotype reminiscent of Th2 cells. First, several studies have linked TGFβ signaling to the inhibition of Th2 differentiation, through direct inhibition of GATA3 expression^45^. Given the observed increase in GATA3 expression in Pak2-deficient Tregs, it is possible that Pak2 might promote TGFβ signaling, either through regulating expression of key components of the pathway or directly transducing signals downstream of the TGFβ receptor (TGFβR). In support of this hypothesis, a dominant negative form of Pak2 inhibited TGFβ-induced transformation and proliferation of fibroblast cells^46^, suggesting that Pak2 may play an active role in modulating TGFβ signaling. Second, it is possible that Pak2 might directly remodel chromatin at specific gene loci. Within *in vitro* cultures, Pak2 was shown to phosphorylate a serine residue on the H4 nucleosomal component of 293T cells^47^. Phosphorylation at this site enhanced the formation of permissive H3.3-H4 nucleosomes while simultaneously inhibited the formation of H3.1-H4 nucleosomes associated with gene silencing^47^. Therefore, it is possible that Pak2 may directly modulate the Treg transcriptome by permitting active chromatin at Treg-associated genes while concomitantly silencing expression of Th2-associated genes. Both of these possibilities warrant further investigation.

Collectively, we found that Pak2 is essential for Treg suppressive function through maintaining expression of key Treg functional molecules and by protecting Tregs from losing their suppressive phenotype and becoming pathologic Th2 cytokine-secreting cells. Although the mechanism by which Pak2 serves as a signaling intermediate in regulating the balance between Treg immunosuppression and immunopathology warrants further investigation, these insights would be therapeutically significant for multiple diseases, including Th2-mediated allergic hypersensitivity, autoimmunity and tumor immunology.

## Methods

### Mice

Mice were bred and used under the animal study protocol approved by the Northwestern University Animal Care Use Committee. All mice were housed in the specific pathogen-free facility at Northwestern University according to the university and National Institute of Health guidelines. The generation of *Pak2*-floxed mice (*Pak2* ^*F/F*^) has been previously described^48^. To generate Treg-specific Pak2-deficient mice, *Pak2* ^*F/F*^ mice were crossed with mice expressing the Cre-recombinase under the control of the *Foxp3* promoter^23^. C57BL/6 mice with the CD45.1^+^ congenic marker (B6.SJL-*Ptprc* ^*a*^ *Pepc* ^*b*^/BoyJ) that were used for *in vitro* Treg suppression assays were originally obtained from the Jackson Laboratory.

### Tissue dissociation and cell isolation

To obtain thymocytes or T cells, thymi or peripheral lymphoid organs (spleen, peripheral/mesenteric lymph nodes) were dissociated through a 70-μm nylon mesh into RPMI (supplemented with 10% FBS). Cells were washed with FACS buffer (2% FBS, 2% NaN_3_, 2 mM EDTA) and passed through a 40- μm nylon mesh prior to antibody staining.

### Flow cytometry and antibodies

Flow cytometric analyses were done at the Robert H. Lurie Flow Cytometry Core Facility (RHLCCC) using the Fortessa (BD) flow cytometry system. For surface staining, cells were incubated with fluorochrome-conjugated antibodies for 30 minutes at 4 °C using 1:200 dilutions of each antibody (unless otherwise specified). Cells were washed twice in FACS buffer prior to being analyzed or stained intracellularly using the Foxp3/Transcription Factor Staining Buffer Set (eBioscience). Dead cells were excluded either using DAPI (Life technologies) or LIVE/DEAD® Fixable Dead Cell Stain Kits (Life technologies). Forward and side scatter was used to identify live lymphocytes. Data analysis was performed using FlowJo (version 9.6.2) software (Tree Star). Antibodies against mouse CD4 (GK1.5), anti-CD8α (53–6.7), anti-CD62L (MEL-14), anti-GATA3 (L50–823) and anti-CD44 (IM7) were from BD Biosciences. An antibody against mouse CD25 (PC61) was from BioLegend. Antibodies against mouse GITR (DTA-1), anti-CTLA4 (UC10–4B9), anti-Foxp3 (FJK-165), anti-IFNγ (XMG1.2), anti-IL17A (eBio17B7), anti-IL4 (11B11), anti-RORγt (B2D), anti-T-bet (eBio4B10) and anti-CD69 (H1.2F3) were from eBioscience. An antibody against mouse Neuropilin-1 was from R&D Systems. CellTrace™ CFSE Cell Proliferation Kit (Thermo Scientific) for flow cytometry was used to track cell proliferation in *in vitro* Treg suppression assays.

### Tissue histology

To analyze peripheral tissue inflammation in *Pak2* ^*F/F*^;*Foxp3*-Cre mice, the Mouse Phenotyping and Histology Laboratory (MPHL) at Northwestern University performed a comprehensive organ necropsy, including hematoxylin and eosin (H&E) staining of the thymus, spleen, lymph nodes, liver, heart, pancreas, salivary glands, kidneys, esophagus, stomach and intestines. For acquisition of H&E staining images, an Olympus BX41 Stereo Compound Microscope with UPLFLN 4X, 10X and 40X objectives with air at 25 °C was used. NIS-D Documentation Imaging software was used as acquisition software.

### *In vitro* stimulation and cytokine staining

Cells isolated from the spleen and lymph nodes (10 × 10^6^ cells seeded at a concentration of 5 × 10^6^ cells/ml) from *Pak2* ^*F/F*^ (WT) and *Pak2* ^*F/F*^;*Foxp3*-Cre (KO) mice were stimulated in a 6-well plate with 50 ng/ml PMA (Sigma Aldrich) and 500 ng/ml ionomycin (Sigma Aldrich) in complete medium (RPMI 1640, 10% FBS). Cells were stimulated for a total of 4 hours prior to staining for flow cytometry. GolgiStop (BD Biosciences) was added to the culture medium for the final 3 hours of stimulation. Cells were then washed twice in FACS buffer (2% FBS, 2% NaN_3_, 2 mM EDTA) and stained for flow cytometry as previously described.

### Serum cytokine analysis

Blood was collected from *Pak2* ^*F/F*^ (WT) and *Pak2* ^*F/F*^;*Foxp3*-Cre (KO) mice by retro-orbital bleeding, allowed to clot for 2 hours at room temperature and the serum aspirated following centrifugation at 3000 × g for 10 minutes. Serum cytokine levels of GM-CSF, IFN-γ, IL-2, IL-4, IL-5, IL-6, IL-10, IL-13, IL-17, TNF-α, M-CSF and G-CSF were determined using a MILLIPLEX MAP Mouse Cytokine/Chemokine Magnetic Bead Panel (EMD Millipore) using the MAGPIX® System (EMD Millipore) and the data analyzed using Milliplex® Analyst 5.1 software.

### Serum immunoglobulin and autoantibody detection

Blood was collected, as previously described, and analyzed for serum immunoglobulins (IgM, IgG1, IgG2c, IgG2b, IgG3, IgA and IgE) using the C57BL/6 Mouse Immunoglobulin Panel (Southern Biotech), following manufacturer’s instructions. Serum levels of anti-nuclear antigens were determined using the Mouse Anti-Nuclear Antigens (ANA/ENA) Ig’s (total (A+G+M)) ELISA Kit (Alpha Diagnostics International), following manufacturer’s instructions.

### *In vitro* Treg suppression assay

CD4+ T cells were isolated from the spleen and lymph nodes (brachial, axial, inguinal and mesenteric) of CD45.2 *Pak2* ^+/+^;*Foxp3*-Cre (WT) and *Pak2* ^*F/F*^;*Foxp3*-Cre (KO) mice by magnetic depletion using the Easy Sep CD4 Isolation Kit (Stemcell Technologies). Using these cells, we sorted WT and Pak2-deficient CD4+YFP+ Tregs. Simultaneously, CD4+CD25−CD45RB^hi^ naïve T cells were sorted from WT CD45.1 mice. Naïve T cells were stained with 5 μM carboxyfluorescein succinimidyl ester (CFSE) for 8 minutes at room temperature. In combination with 1x10^5^ irradiated splenocytes and 1 μg/ml anti-CD3, 4x10^4^ CD4+CD25−CD45RB^hi^ naïve T cells were incubated with varying ratios of CD4+YFP+ Tregs derived from WT or KO mice (Ratio of naïve:Treg; 1:0, 1:1, 1:2, 1:4, 1:8, 1:16, 1:32) in complete mouse media. Cells were incubated for 72 hours at 37 °C prior to harvesting. To separate out CFSE-stained T cells from unstained APCs and Tregs, cells were stained with anti-CD45.1 and CFSE dilution was monitored by flow cytometry.

### *In vivo* Treg suppression assay

CD4+ T cells were isolated from the spleen and lymph nodes (brachial, axial, inguinal and mesenteric) from *Pak2* ^+/+^;*Foxp3*-Cre (WT) and *Pak2* ^*F/F*^;*Foxp3*-Cre (KO) mice by magnetic depletion using the Easy Sep CD4 Isolation Kit (Stemcell Technologies). Using these cells, we sorted WT and Pak2-deficient CD4+YFP+ Tregs. Wild-type CD4+CD25−CD45RB^hi^ naïve T cells were simultaneously sorted. A total of 4x10^5^ naïve T cells were transferred into Rag1^−/−^ mice via tail vein injection, either alone or in combination with 2 x10^5^ WT Tregs or 2x10^5^ Pak2 KO Tregs. Mice were monitored weekly for weight loss and sacrificed following loss of more than 20% body weight.

### RNA isolation and cDNA microarray

CD4+ T cells were isolated from the spleen and lymph nodes (brachial, axial, inguinal and mesenteric) by magnetic depletion using the Easy Sep CD4 Isolation Kit (Stemcell Technologies). CD4+YFP+ Tregs from *Pak2* ^+/+^;*Foxp3*-Cre (WT) and *Pak2* ^*F/F*^;*Foxp3*-Cre (KO) mice were sorted and RNA isolated using the RNeasy Micro Kit (Qiagen). RNA concentrations were determined using the NanoDrop 2000 and RNA quality was assessed using the Agilent 2100 Bioanalyzer (Agilent Technologies). Only samples with an RNA integrity number (RIN) above 9 were used for cDNA synthesis and microarray analysis. cDNA was generated using the Illumina® TotalPrep™ RNA Amplification Kit (ThermoFisher Scientific) according to the manufacturers instructions. Microarray analysis was performed using the multi-sample format MouseWG-6 v2.0 Expression BeadChip (Illumina) and analyzed using the iScan Array Scanning System (Illumina). R statistical software was used to generate heat maps. Ingenuity pathway analysis (IPA) (Qiagen) was used to determine the diseases, functions, networks and pathways that were altered between *Pak2* ^+/+^;*Foxp3*-Cre (WT) and *Pak2* ^*F/F*^;*Foxp3*-Cre (KO) Tregs.

### Treg stimulation and cytokine analysis

Cells were isolated from the spleen and peripheral lymph nodes (brachial, axial and inguinal) of *Pak2* ^+/+^;*Foxp3*-Cre (WT) and *Pak2* ^*F/F*^;*Foxp3*-Cre (KO) mice and Tregs sorted based on YFP expression. WT and KO Tregs were seeded into a round-bottom 96-well plate at a concentration of 1 × 10^6^ cells/ml and incubated in either unstimulated or stimulated conditions for 72 hours at 37 °C (5% CO_2_). For unstimulated conditions, sorted Tregs were resuspended in complete RPMI-1640 media supplemented with IL-2 (50 U/ml) to maintain cell viability. For stimulated conditions, wells were coated with anti-CD3 (10 μg/ml) overnight at 4 °C in sterile PBS. Sorted Tregs were resuspended in complete RPMI-1640 media supplemented with soluble anti-CD28 (2 μg/ml) and IL-2 (50 U/ml). Following 72 hours stimulation, the supernatant was removed and cytokine levels of IFN-γ, IL-4, IL-5, IL-10, IL-13 and IL-17 were determined using a MILLIPLEX MAP Mouse Cytokine/Chemokine Magnetic Bead Panel (EMD Millipore) using the MAGPIX® System (EMD Millipore) and the data analyzed using Milliplex® Analyst 5.1 software.

### Statistical analysis

Statistical analysis and graphs were generated using Prism 6 (GraphPad Software).

## Electronic supplementary material

Supplementary Figures
