# Supplementary material for: Pak2 is essential for the function of Foxp3+ regulatory T cells through maintaining a suppressive Treg phenotype
Source: Sci Rep. 2017 Dec 6;7:17097. doi: 10.1038/s41598-017-17078-7 (PMC5719048; doi:10.1038/s41598-017-17078-7)
Supplement: Supplementary file 1 — Supplementary Figures [file 41598_2017_17078_MOESM1_ESM.pdf]

Pak2 is essential for the function of Foxp3+ regulatory T cells through maintaining a suppressive Treg phenotype.

Kyle L. O'Hagan[1], Stephen D. Miller[1], Hyewon Phee[1,2]

[1]Department of Microbiology-Immunology

Feinberg School of Medicine

Northwestern University, Chicago, IL 60611

[2]Immuno-Oncology Research

Amgen, Inc.

South San Francisco, CA, 94080

Correspondence should be addressed to H.P. (hyewonp@amgen.com)

Hyewon Phee, PhD.

Amgen, Inc.

1120 Veterans Boulevard

South San Francisco, CA 94080

Tel: 415-317-5431

Supplementary Figures

Supplementary Fig 1. O'Hagan et al.

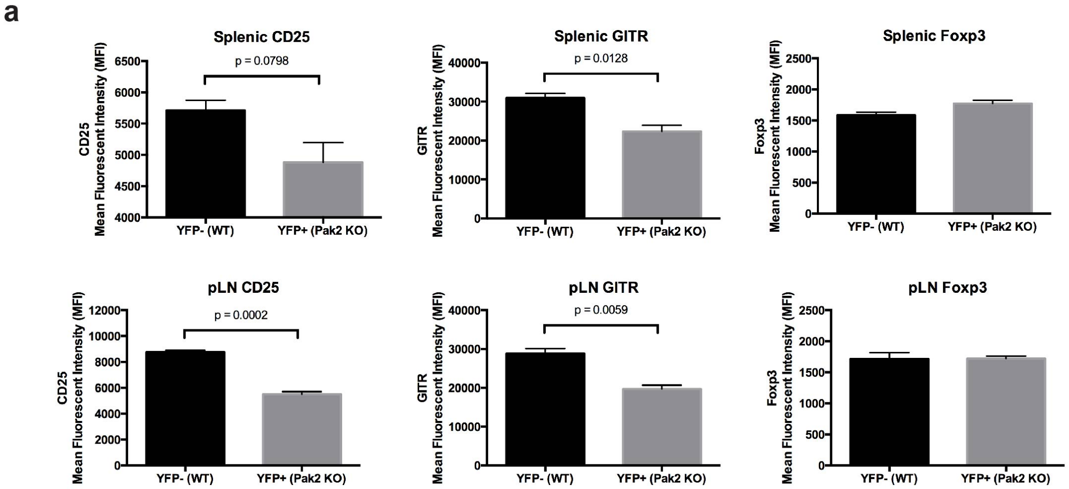

Supplementary Figure 1: Loss of Foxp3 expression in homozygous *Foxp3*-Cre Pak2 KO mice is not cell intrinsic.

(a) Flow cytometric analysis of CD25, GITR and Foxp3 expression within Tregs from the spleen (*upper panel*) and peripheral lymph nodes (pLN; *lower panel*) of male WT and *Foxp3*-Cre Pak2 KO mice. (b) Flow cytometric analysis of CD25, GITR and Foxp3 expression within Tregs from the spleen (*upper panel*) and peripheral lymph nodes (pLN; *lower panel*) of homozygous female WT and *Foxp3*-Cre Pak2 KO mice. (c) Mean fluorescent intensity (MFI) of CD25, GITR and Foxp3 expression within WT and *Foxp3*-Cre Pak2 KO Tregs derived from the spleen (*upper panel*) and pLN (*lower panel*) of heterozygous female *Pak2*$^{F/F}$;*Foxp3*-Cre+/− mice. Graphs within this figure show mean ± SE and *p*-values are indicated for significant differences. Results are representative of at least three individual replicates.

Supplementary Fig 2. O'Hagan et al.

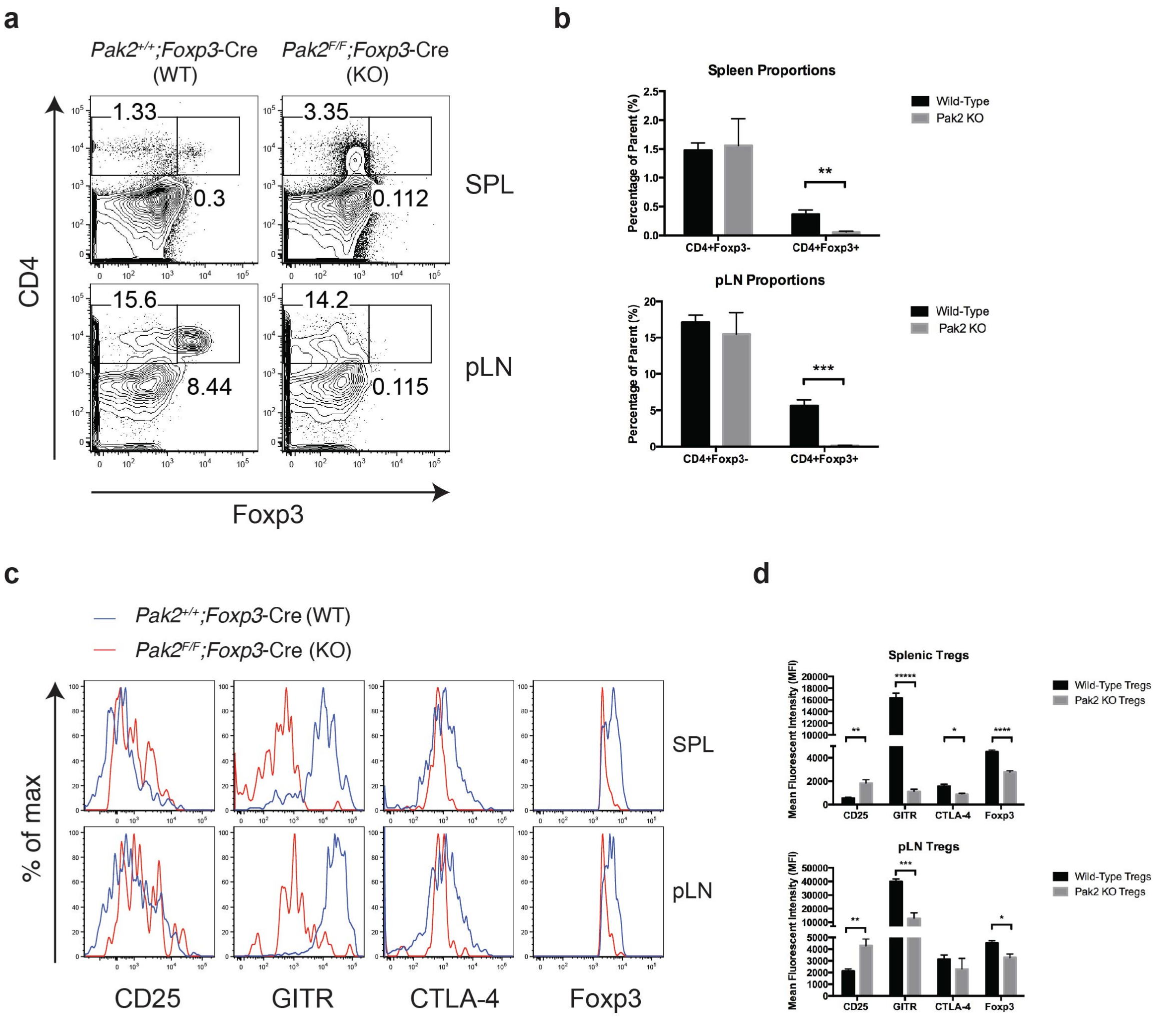

Supplementary Figure 2: Loss of Pak2-deficient Foxp3+ Tregs upon transfer to immunodeficient hosts.

(a) Flow cytometric analysis of CD4 and Foxp3 expression within total live cells from the spleen (SPL) and peripheral lymph nodes (pLN) from Rag1-deficient hosts that had received either YFP+ WT or *Foxp3*-Cre Pak2 KO Treg cells. (b) Proportion of CD4+Foxp3− T cells (*left bars*) and CD4+Foxp3+ Treg cells (*right bars*) from the spleen (*upper graph*) and the pLN (*lower graph*) of Rag1-deficient hosts that had received either YFP+ WT or *Foxp3*-Cre Pak2 KO Treg cells. (c) Flow cytometric analysis of CD25, GITR, CTLA-4 and Foxp3 expression within

Foxp3+ Tregs from the spleen (*upper panel*) and pLN (*lower panel*) derived from Rag1-deficient hosts that had received either YFP+ WT (*blue histogram*) or *Foxp3*-Cre Pak2 KO (*red histogram*) Treg cells. (d) Mean fluorescent intensity (MFI) of CD25, GITR, CTLA-4 and Foxp3 expression within Tregs derived from Rag1-deficient hosts that had received either YFP+ WT or *Foxp3*-Cre Pak2 KO Treg cells. Results are shown for the spleen (*upper graph*) and the pLN (*lower graph*). Graphs within this figure show mean ± SE (n=3). *0.01 < p < 0.05, **0.001 < p < 0.01, ***0.0001 < p 0.001, ****0.00001 < p < 0.0001, *****0.00001 < p < 0.000001 (unpaired two-tailed Student t test). Results are representative of at least three independent experiments.

Supplementary Fig 3. O'Hagan et al.

a

Top Diseases and Biological Functions	p-value
Immunological Disease	2.16E-04 - 4.43E-18
Inflammatory Response	1.99E-04 - 1.21E-14
Endocrine System Disorders	1.39E-04 - 4.72E-12
Gastrointestinal Disease	1.50E-04 - 4.72E12
Metabolic Disease	2.06E-06 - 4.72E-12

b

Top Canonical Pathways	-log[p-value]
Altered T Cell and B Cell Signaling in Rheumatoid Arthritis	4.83E+00
Crosstalk between Dendritic Cells and Natural Killer Cells	3.43E+00
Autoimmune Thyroid Disease Signaling	3.40E+00
T Helper Cell Differentiation	3.15E+00
STAT3 Pathway	3.09E+00
Nur77 Signaling in T Lymphocytes	2.78E+00
PTEN Signaling	2.68E+00
IL4 Signaling	2.26E+00

c

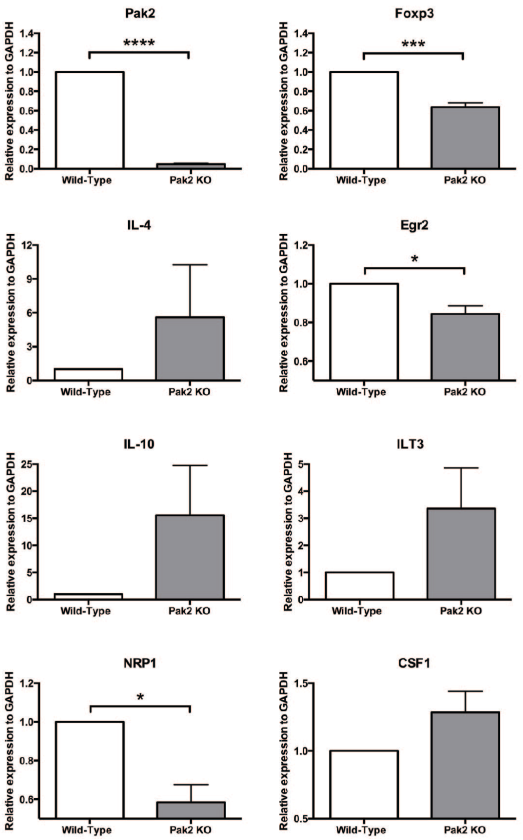

d

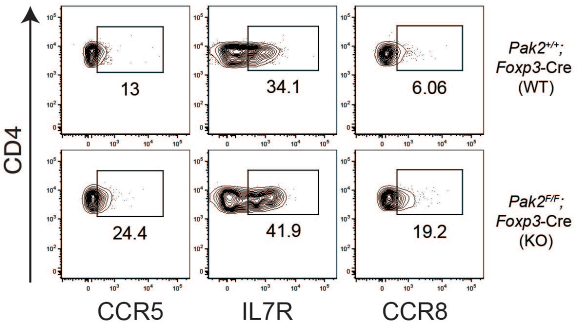

Supplementary Figure 3: Confirmation of up- and down-regulated genes in Pak2-deficient Tregs.

(**a**) Ingenuity Pathway Analysis (IPA) of the top diseases and biological functions that were altered in Pak2-deficient Tregs relative to WT Tregs. (**b**) IPA of the top canonical pathways that were altered in Pak2-deficient Tregs relative to WT Tregs. (**c**) mRNA levels of *Pak2*, *Foxp3*, *Il4*, *Egr2*, *Il10*, *Lilrb4* (ILT3), *Nrp1* and *Csf1* in Tregs derived from *Pak2*$^{F/F}$ (WT) and *Pak2*$^{F/F}$;*Foxp3*-Cre (KO) mice, as determined by real-time polymerase chain reaction (RT-PCR) and normalized to GAPDH expression. (**d**) Flow cytometric analyses of the expression of CCR5, IL7R and CCR8 within CD4+Foxp3+ Tregs from the pLN of WT and Foxp3-Cre KO mice. Graphs within this

figure show mean ± SE (n = 3 for (**a, b**), n = 3 for (**c**), n = 2 for (**d**)). *0.01 < p < 0.05, ***0.0001 < p 0.001, ****0.00001 < p < 0.0001 (unpaired two-tailed Student t test).
